# Case Report: Novel Compound Heterozygotic Variants in *PPP2R3C* Gene Causing Syndromic 46, XY Gonadal Dysgenesis and Literature Review

**DOI:** 10.3389/fgene.2022.871328

**Published:** 2022-06-23

**Authors:** Wei Zhang, Jiangfeng Mao, Xi Wang, Bang Sun, Zhiyuan Zhao, Xiaoxia Zhang, Min Nie, Xueyan Wu

**Affiliations:** ^1^ NHC Key Laboratory of Endocrinology (Peking Union Medical College Hospital), Department of Endocrinology, Peking Union Medical College, Chinese Academy of Medical Sciences, Beijing, China; ^2^ State Key Laboratory of Complex Severe and Rare Diseases, Peking Union Medical College Hospital, Peking Union Medical College, Chinese Academy of Medical Sciences, Beijing, China

**Keywords:** disorders/differences of sex development(DSD), syndromic 46 XY DSD, gonadal dysgenesis, facial deformity, *PPP2R3C* mutations, compound heterozygous variants

## Abstract

**Purpose:** Patients with syndromic 46, XY disorders/differences of sex development (DSD) are characterized by gonadal and phenotypic genders inconsistent with their chromosomal sexes as well as abnormalities of multiple extragonadal organs. They are caused by mutations in specific genes, which are expressed in the affected organs and regulate their development, and over fourteen genes have been identified. In this study, we aimed to determine the underlying cause of a patient with syndromic 46, XY DSD and review the clinical presentations and genetic findings of all reported similar cases.

**Methods:** Whole-exome sequencing (WES) was performed to find a molecular cause of the patient. *In silico* tools were used to analyze the pathogenicity of the variants. Reports of cases with similar clinical features and involved genes were summarized by searching through PubMed/MEDLINE using keywords “*PPP2R3C*” or “*G5PR*” and “46,XY disorders of sex development”.

**Results:** Compound heterozygous variants (p.F229del/p.G417E) in *PPP2R3C* were identified in the 24-year-old female by WES and verified by Sanger sequencing. The patient presents complete testicular dysgenesis, low birth weight, facial deformity, cubitus valgus, and decreasing number of CD19^+^ B lymphocytes and CD4^+^ T lymphocytes. A total of thirteen 46, XY DSD cases with four homozygous *PPP2R3C* mutations (p.Leu103Pro, p.Leu193Ser, p.Phe350Ser, and p.Ser216_Tyr218dup) have been reported previously, and their clinical manifestations are roughly similar to those of our patient.

**Conclusion:** Novel compound heterozygous variants in *PPP2R3C* cause specific syndromic 46, XY gonadal dysgenesis, which broadened the pathogenic variants spectrum of *PPP2R3C*. The typical phenotype of *PPP2R3C* mutation is complete 46, XY gonadal dysgenesis with multiple extragonadal anomalies, including facial deformities, skeletal system abnormalities, muscle abnormalities, impaired nervous system, impaired hearing and vision, heart and kidney anomalies, and gastrointestinal dysfunction.

## Introduction

46, XY disorders/differences of sex development (DSD) is a condition in which phenotypic and/or gonadal sex do not match with 46, XY chromosomal sex. The disease may present a dysgenetic gonadal, testicular, ovotesticular, or ovarian tissue and variable degrees of virilization of the external genitalia, along with structures derived from the Wolffian or Müllerian duct. According to the different pathogenesis, 46, XY DSD is mainly divided into gonadal development abnormalities, insufficiency of androgen synthesis, insensitivity to androgens, or Müllerian duct persistent syndrome (Bashamboo and McElreavey, 2015). The etiology of 46, XY DSD is mutations of genes involved in androgen synthesis, androgen action, or Müllerian duct regression. Currently, more than 35 genes have been identified as genetic causes of 46, XY DSD (Baxter et al., 2015; Yu et al., 2021).

46, XY DSD can be classified as either syndromic or non-syndromic 46, XY DSD based on the presence or absence of extragonadal abnormalities (Hiorta and Gillessen-Kaesbachb, 2009). The incidence of syndromic 46, XY DSD is very low and usually caused by mutations in specific genes that play essential roles in both sex development and extragonadal organ formation. More than 14 genes at present are known to be linked with syndromic 46, XY DSD, such as *WT1*, *NR5A1*, *SOX9*, *FGFR2*, *HOXA13*, *DHCR7*, and *XH2*, most of which are genes encoding transcript factors (Bagheri-Fam et al., 2015; Tamura et al., 2017).

Sex-determining region Y (SRY)-box 9 (SOX9) protein, a very important transcript factor, regulates the onset, development, and maintenance of the testis formation, and it is also essential for the early chondrogenesis in humans. Mutations or deletions of *SOX9* can cause a syndromic 46, XY DSD, presenting as 46, XY sex reversal and campomelic dysplasia. The SOX9-phosphoprotein can be translocated into the nucleus of Sertoli cells and maintains differentiation of Sertoli cells (Malki et al., 2005). Human *PPP2R3C* (Protein Phosphatase 2 Regulatory Subunit B Gamma gene, OMIM: 615902) is located on 14q13.2, which has 13 exons encoding a 453-amino acid protein. Recently, it was found that the *PPP2R3C* gene regulates the balance of phosphorylation and dephosphorylation of Sox9 protein by forming a protein phosphatase complex. The mutation of the *PPP2R3C* gene is identified as a novel genetic etiology for syndromic 46, XY DSDs (Guran et al., 2019). Homozygous variants in the *PPP2R3C* gene can cause a syndromic 46, XY DSD, which manifests specific facial features, myopathy, musculoskeletal abnormalities, and neural motor delay.

In this study, we described the clinical, laboratory characteristics, and novel compound heterozygous *PPP2R3C* variants in a Chinese patient diagnosed with 46, XY gonadal dysgenesis. The clinical presentations and genetic findings of similar cases reported previously were also reviewed to improve our understanding of the phenotypes and genotypes in patients with *PPP2R3C* mutations.

## Methods

### Whole-Exome Sequencing

Patient’s genomic DNA was extracted from 200 μl peripheral blood leukocytes using the Qiagen DNA Blood kit (Qiagen, Dusseldorf, Germany). First, 50 ng DNA was broken into a size of about 200bp by fragmentation enzymes. The DNA fragments were then end-repaired, and the 3’end was added with one A base. Second, the DNA fragments were ligated with barcoded sequencing adaptors, and fragments of about 320bp were collected by XP beads. After PCR amplification, the DNA fragments were hybridized and captured by NanoWES according to the manufacturer’s protocol. The hybrid products were eluted and collected and then subjected to PCR amplification and purification. Next, the libraries were quantified by qPCR, and size distribution was determined using an Agilent Bioanalyzer 2100 (Agilent Technologies, Santa Clara, CA, United States). Finally, the Novaseq6000 platform (Illumina, San Diego, United States), with 150 bp pair-end sequencing mode, was used for sequencing the genomic DNA of the patient. Raw image files were processed using CASAVA v1.82 for base calling and generating raw data. The sequencing reads were aligned to the human reference genome (hg19/GRCh37) using the Burrows–Wheeler Aligner tool. GATK software was employed for variant calling. Variant annotation and interpretation were conducted by ANNOVAR.

### TA Clone and Sanger Sequencing

Exon 7 and exon 13 of the *PPP2R3C* gene were amplified by PCR, and then the PCR products of exon 7 and exon 13 were purified and underwent Sanger sequencing. Primers were designed using Primer3 software (http://www.bioinformatics.nl/cgi-bin/primer3plus/primer3plus.cgi). Primer sequences were as follows: PPP2R3C-E7-F: 5′-ACT​ACA​TAT​TGG​AAC​TTA​TCC​CTA​CG-3′, PPP2R3C-E7-R: 5′-GGCAG TGAC AGG​AAA​ATA​AAT​GAT​A-3′; PPP2R3C-E13-F: 5′-ATT​TTA​TTC​CTC​GTA​CTT​CAG TGGA-3′, PPP2R3C-E13-R: 5′-GGTCCTATT GCTTCTAAACTATT GC -3′.

TA clone of exon 7 was performed for a small deletion existing in one allele of the patient. The Taq polymerase-amplified PCR products of exon 7 were inserted into the pMD19T vector, and then the constructs were transformed into DH5α-competent cells and grew in the X-Gal and IPTG plates. The white clones were selected and amplified, and their DNA was extracted and sequenced.

Sequencing of PCR products and TA clone was performed using a Taq big dye terminator sequencing kit and an ABI-3730 automated sequencer (Applied Biosystems, Foster City, CA, United States). DNAs from both parents were sequenced to ascertain the inheritance. We read the sequencing results by 4Peaks (Nucleobytes, Netherlands) and then blasted each in NCBI (https://blast.ncbi.nlm.nih.gov/Blast.cgi) taking the following sequence as references: cDNA: NM_017917.4; protein: NP_060387.2.

### Three-Dimensional Visualization and Prediction

Structural models of wild PPP2R3C protein and mutant protein were built by inputting protein sequences in the SWISS-MODEL server (https://swissmodel.expasy.org). Then, we downloaded the PDB file and uploaded it into UCSF ChimeraX (version: 1.2.5), a molecular visualization program. By comparing the changes in the contacts and hydrogen bonds with other amino acids in a spatial model, we can predict the potential pathogenic effects the specific variant brings to the protein.

### Clinical Evaluation

Skeletal deformity and external genital development were assessed by physical examination. Uterine and gonadal development was assessed by laparoscopy. Bone age was assessed by X-rays of hands. In addition, we evaluated the muscular and lymphocytic systems using electromyography and lymphocyte counts. Hormone test results included levels of serum follicle-stimulating hormone (FSH), luteinizing hormone (LH), and testosterone (T), which were measured by chemiluminescent immunoassays (Bayer Diagnostics Corporation, United States).

### Literature Review

We searched for reports about *PPP2R3C* variations in PubMed and MEDLINE, and the keywords for searching included “PPP2R3C” or “G5PR” in combination with “46,XY disorders of sex development”. Available data on clinical evaluations and genetic findings were extracted and summarized.

## Results

### Compound Heterozygous Variants in *PPP2R3C*

**FIGURE 1:**
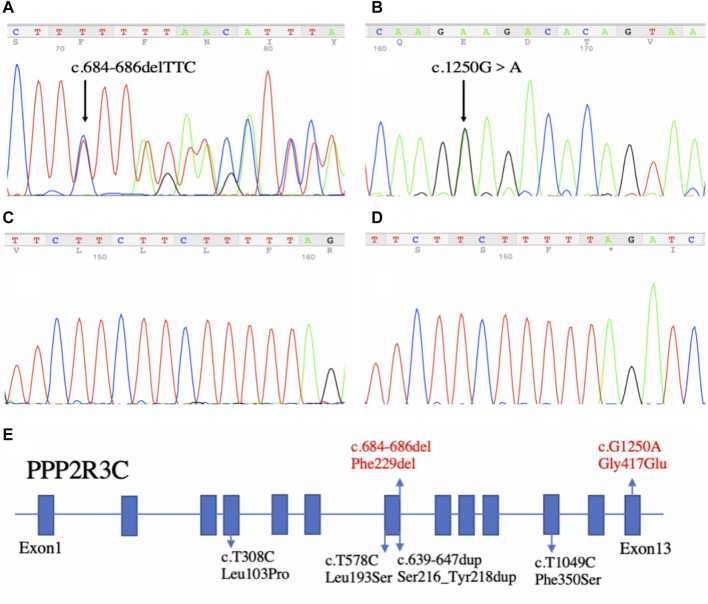
Sequencing results of our patient and the summary of *PPP2R3C* mutations found in patients with 46, XY disorders/differences of sex development. **(A)** In-frame deletion (c.684_686delTTC) in exon 7 and **(B)** missense mutation (c.1250G > A) in exon 13. **(C,D)** Sequencing results of TA-clone. Left panel **(C)**: wild-type allele; Right panel **(D)**: other allele with the in-frame deletion (c.684_686delTTC). **(E)** Summary of *PPP2R3C* mutations found in patients with 46, XY disorders of sex development. Blue boxes indicate the exons of *PPP2R3C*. Four reported variants are depicted in black, and the novel compound heterozygotic variants are depicted in red.

**FIGURE 2:**
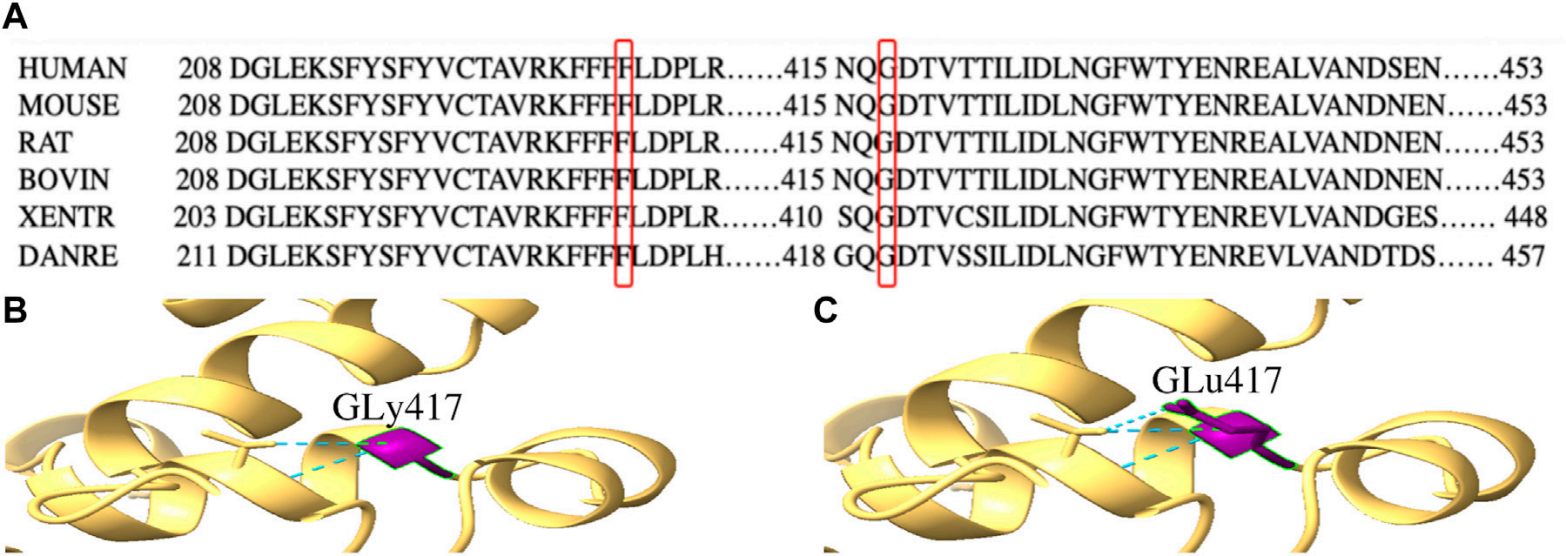
Conservation analysis of *PPP2R3C* 229F and 417G and three-dimensional molecular models of p.G417E PPP2R3C. **(A)** Partial alignment of *PPP2R3C* amino acid sequence, showing high conservation of phenylalanine (at position 229) and glycine (at position 417); **(B,C)** Gly417 and Glu417 residues are depicted in purple. The mutation of p.G417E will change the number of hydrogen bonds (blue).

By WES, we identified two variants (c.684_686delTTC/p.F229del in exon 7 and c.1250G > A/p. G417E in exon 13) of *PPP2R3C* and confirmed by Sanger sequencing (Figure 1) in the patient, which came from his father and mother, respectively. Potentially pathogenic variants in other genes associated with syndromic 46, XY DSD had not been found. The two wild-type amino acids involved in both variants are highly conserved among ortholog proteins (Figure 2). The deletion of Phe229 will cause an incomplete alpha helix structure and change the condition of four repeated phenylalanines. The Gly417Glu does not affect a significant protein domain, but the number of hydrogen bonds and contacts formed within residues is changed (Figure 2).

### Clinical Manifestation

A 24-year-old female of Chinese Han descent was diagnosed with 46, XY gonadal dysgenesis at age 18, when she was presented with poor development of secondary sexual characteristics and primary amenorrhea. She was born of a non-consanguineous marriage at term by vaginal delivery without any perinatal complications and was raised female gender. She was born with a birth weight of around 1 kg and normal body length. There was no history of feeding difficulties or developmental retardation in childhood. At ten years of age, she presented retardation of bone age about two years, and the closure of bone epiphysis did not appear till over 20 years old. The patient was 148 cm tall (-3SD) at 18 years old and was diagnosed with short stature. She received growth hormone therapy for three months and gained the height increase of about 2.5 cm in the following two years. The patient did not receive other therapy till presentation.

**FIGURE 3:**
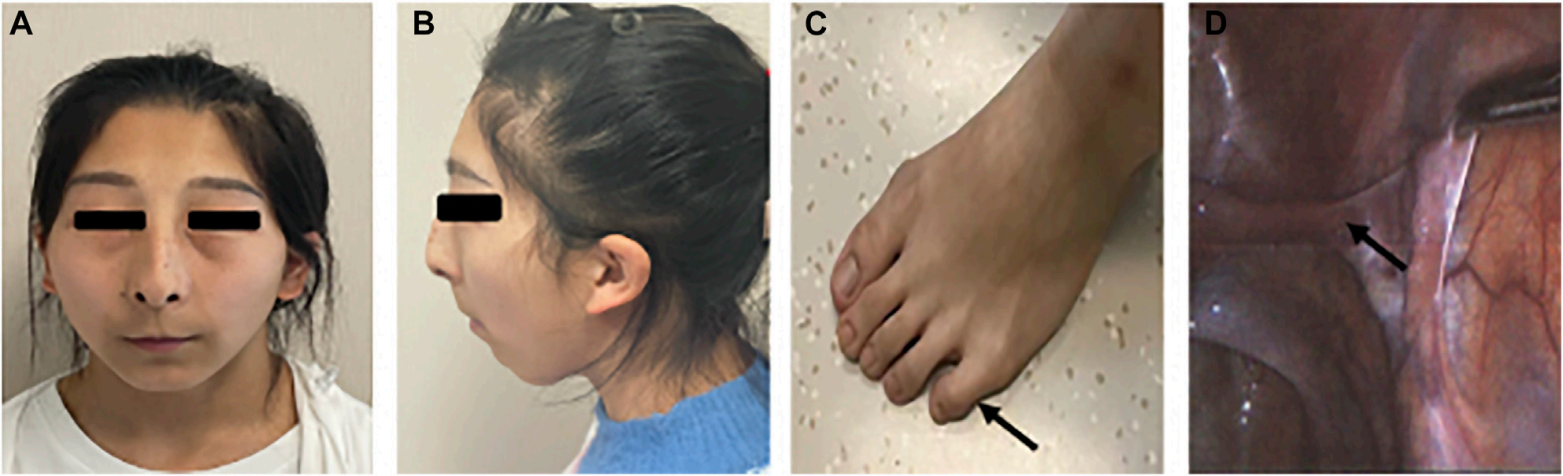
Partial clinical presentations of the patient. **(A)** Facial frontal photo of the patient and **(B)** facial profile photo of the patient: flat face, thin lips, low ears, mandibular retrusion, dysplasia of the alar nose, **(C)** short fifth phalanx, and **(D)** oviduct-like structure in laparoscopy.

On physical examination, she had a height of 155 cm, a weight of 48 kg, and a blood pressure of 124/75 mmHg. Tanner staging was B1P2. External genitalia revealed the clitoris of normal and puerile female genitalia. There were two distinct openings in the urethra and vagina. No gonads or lumps were found in the inguinal region. The patient did not have facial hair, acne, male torso, body hairs, and deepening of voice. Systemic examination revealed remarkable facial features including unruly scalp hair, thin eyebrows, thin lips, and long and smooth philtrum. The patient had hypoplastic ala nasi and beaked nose, low-set ears, and facial pigmented nevus which were non-ignorable facial characteristics as well. The systemic examination also revealed abnormalities of the musculoskeletal system including a short fifth phalanx, webbing of the neck, and cubitus valgus (Figures 3A–C).

On investigation, the hormone profile showed LH 23.22IU/L (normal range 1.2–8.6), FSH 70.88IU/L (normal range 1.2–19.2), T 0.34 ng/ml (normal range 1.75–7.81), E2 11 pg/ml (normal range <39), 17-OHP 1.19 ng/ml (-), FT4 1.19 ng/dl (normal range 0.81–1.89), FT3 2.95 pg/ml (normal range 1.80–4.10), and TSH1.2μIU/ml (normal range 0.38–4.34) (Table.1). Lab evaluation also included a normal white blood cell count, normal hemoglobin concentration, and normal lymphocyte count and proportion. *PPP2R3C* gene is highly expressed in lymphocytes and plays a role in the survival of CD19^+^ B cells. Gene knockout (PPP2R3C-/-) mice by conditional targeting in CD19^+^ B cells showed a deficit in B-cell survival and a reduced number of mature B cells. After we identified the P*PP2R3C* variants of our patient, we tested her T/B cell subsets. The analysis of T/B cell subsets revealed a decreased number of CD19^+^ B cells (1.6%, normal range: 8.5%–14.5%) and CD4^+^ T cells (21.5%, normal range: 30.0–46.0%) (Table.2). The karyotype was 46, XY. Ultrasound and laparoscopy revealed the dysgenesis of the uterus and bilateral oviduct-like structure (Figure 3D).

**TABLE 1:** Sex hormones of the patient.

Hormone	Normal range (males)	Before gonadectomy	After gonadectomy (with estrogen therapy)
FSH(IU/L)	1.2–19.2	70.9	24.4
LH(IU/L)	1.2–8.6	23.2	15.9
T(ng/ml)	1.75–7.81	0.34	0.46
E2(pg/ml)	<39	11	53

**TABLE 2:** Lymphocyte subsets of the patient.

Immune cells	Count (/L)	Percentage (%)	Normal range
CD19^+^ B cells	3.04 × 10^7	1.6	8.5∼14.5%
NK	6.14 × 10^8	32.3	9.5∼23.5%
T	1.22 × 10^9	64.3	62.6∼76.8%
CD4^+^ T cells	4.08 × 10^8	21.5	30.0∼46.0%
CD8^+^ T cells	4.96 × 10^8	26.1	19.2∼33.6%

Considering the risk of gonadal malignancy, she underwent bilateral gonadectomy at twenty years of age, and streak gonads and bilateral oviducts were found during the operation. Histopathology of gonadectomy material showed an oviduct-like structure bilaterally. After gonadectomy, she was treated with Femoston (estradiol/dydrogesterone: 1/10 mg), with a dose of one tablet daily for almost four years.

### Literature Review

Thirteen 46, XY DSD patients had been reported in three articles previously (Guran et al., 2019; Cicek et al., 2021; Altunoglu et al., 2022). All the patients came from the consanguineous marriage and had been identified as homozygous *PPP2R3C* variants. Eleven out of thirteen 46, XY DSD patients were presented with complete gonadal dysgenesis, complete female vulva with hypoplastic labium major, and hypoplastic uterus. Sex hormone levels were compatible with complete gonadal dysgenesis. One patient presented with partial testicular dysgenesis, and he had ambiguous genitalia, micropenis, and left cryptorchidism. *PPP2R3C* variants are also reported to be associated with syndromic 46, XX DSD. Three such patients were presented with complete gonadal dysgenesis, recognizable facial features, and multiple extragenital malformations. All patients with *PPP2R3C* mutations had some similar extragonadal system manifestations, such as facial deformity (16 of 16,100%), retardation of bone age (15 of 16, 93.7%), and delayed development of the nervous system (14 of 16, 87.5%). The typical facial deformity includes abnormal eyebrows, low small-ears, and dysplasia of the alar nose. Among sixteen patients, more than half of the cases had special clinical manifestations, such as impaired vision (10 of 16, 62.5%), low birth weight (9 of 16, 56.2%), and myopathy (8 of 16, 50%). Symptoms including, renal agenesis (6 of 16, 37.5%), gastrointestinal dysfunction (6 of 16, 37.5%), sensorineural hearing loss (4 of 16, 25%), and cardiac defect (3 of 16, 18.7%) also appeared in some patients (Table 3).

**TABLE 3:** Clinical and molecular findings of 17 DSD patients with *PPP2R3C* mutations.

	Patient	*PPP2R3C* variant	Karyotype	Gonadal phenotype	Birth weight (g)	Facial deformity	Retarded bone age	Neuromotor delay	Myopathy	Sensorineural hearing loss	Impaired vision	Cardiac defect!	Renal agenesis	Gastrointestinal dysfunction
Guran et al. (2019)	p1	p.L193S c.578T > C	46, XY	CGD	1400	+	+	+	+	NA	Rod cone dystrophy	-	-	-
	p2	p.F350Sc.1049T > C	46, XY	CGD	1750	+	+	+	+	+	Rod cone dystrophy	-	-	Omphalocele
	p3	p.L103P c.308T > C	46, XY	CGD	2800	+	+	+	+	+	Rod cone dystrophy	-	+	Diastasis recti and accessory spleen
	p4	p.F350S c.1049T > C	46, XY	CGD	1900	+	+	+	+	-	Rod cone dystrophy	Bicuspid aorta and mild aortic stenosis	+	Anal atresia, pyloric stenosis, and omphalocele
Guran et al. (2021)	p5	p.L193S c.578T > C	46, XX	CGD	1900	+	+	NA	+	-	Rod cone dystrophy	-	+	-
	p6	p.L193S c.578T > C	46, XY	CGD	2200	+	+	+	+	-	Myopia and amblyopia	-	-	-
	p7	p.L193S c.578T > C	46, XY	CGD	2500	+	+	+	+	-	Myopia and amblyopia	-	-	-
	p8	p.L193S c.578T > C	46, XY	PGD	3190	+	+	-	+	-	-	-	-	-
Kayserili et al. (2022)	p9	p.L193S c.578T > C	46, XX	CGD	1800	+	+	+	-	+	Hypermetropia and amblyopia	-	-	-
	p10	p.L193S	46, XY	CGD	2800	+	+	+	-	+	Hypermetropia and amblyopia	-	-	Anterior ectopic anus
		c.578T > C												
	p11	p.L193S	46, XY	PGD	3210	+	+	+	-	-	Hypermetropia	ASD and LPSVC	-	-
		c.578T > C												
	p12	p.L103P c.308T > C	46, XX	CGD	2260	+	+	+	-	-	-	ASD and mild PS	+	Anterior ectopic anus
	p13	p.L193S c.578T > C	46, XY	CGD	3020	+	+	+	-	NA	-	-	+	Omphalocele and malrotated colon
	p14	p.L193S c.578T > C	46, XY	CGD	3700	+	+	+	-	NA	-	-	-	-
	p15	p.S216_Y218dup c.639_647dup	46, XY	CGD	NA	+	+	+	-	NA	-	-	-	-
	p16	p.S216_Y218dup c.639_647dup	46, XY	CGD	2000	+	-	+	-	NA	NA	-	+	-
This report	p17	p.F229del p.G417E	46, XY	CGD	1000	+	+	-	-	-	-	-	-	-

-, absent; +, present; ASD, atrial septal defect; CGD, complete gonadal dysgenesis; DSD, disorders/differences of sex development; LPSVC, left persistent superior vena cava; NA, not available; PGD, partial gonadal dysgenesis; PS, pulmonary stenosis.

A total of four *PPP2R3C* variants had been identified in DSD patients. Specifically, 10 patients had homozygous p.Leu193Ser variant, and 2 of the other 6 patients had homozygous p.Leu103Pro, p.Phe350Ser, or p.Ser216_Tyr218dup variants, respectively. The association between the genotype and phenotype cannot be found based on the current data (Table 3).

## Discussion

In the present study, we reported a syndromic 46, XY DSD due to the compound heterozygous variants in the *PPP2R3C* gene. The variant p.Gly417Glu was not found in gnomAD, ExAC, or 1000 Genomes databases. p.Phe229del was not found in the 1000 Genomes database but showed low frequency in ExAC (0.0000753) and gnomAD (0.000194452) databases. These two variants were predicted to be harmful to protein function by REVEL, an ensemble method for predicting the pathogenicity of variants. So far, four other variants (p.Leu103Pro, p.Leu193Ser, p.Phe350Ser, and p.Ser216_Tyr218dup) in *PPP2R3C* have been reported in cases with similar clinical characteristics. Three missense mutations (p.Leu103Pro, p.Leu193Ser, and p.Phe350Ser) were predicted to be pathogenic according to *in silico* tools, conserved among species, and not found in gnomAD, ExAC, or 1000 Genomes databases. Therefore, the pathogenic variants of *PPP2R3C* cause a syndromic 46, XY complete gonadal dysgenesis in an autosomal recessive inheritance pattern.

46, XY DSD patients with *PPP2R3C* variants manifested gonadal dysgenesis and complete female external genitalia along with the hypoplastic uterus seen in ultrasound or laparoscopy. Extragonadal multisystem abnormalities were also shared in these patients. They were presented with flat faces, poorly developed nasal alars, thin lips, and low ears. The facial abnormalities might be related to dysplasia of cartilage in ears and nose in the early chondrogenesis. The SOX9 protein played a significant role in early chondrogenesis, and it was suggested that pathogenic variants in *PPP2R3C* can impair SOX9 signaling by upregulating the catalytic function of PP2A (Guran et al., 2019). In addition to facial deformities, low birth weight, retardation of bone age, and limited elbow extension also are common characteristics in patients with *PPP2R3C* variants. Previously reported patients did not show any clinical immunodeficiency symptoms, and serum immunoglobulin concentrations and lymphocyte counts were normal. Our patient’s CD19^+^ B lymphocyte counts showed an obvious decrease, which may be due to the decreased viability and increased apoptosis. Gene knockout (PPP2R3C-/-) mice by conditional targeting in CD19^+^ B cells showed a deficit in B-cell survival and low level of mature B cells (Xing et al., 2005). It is suggested that PPP2R3C is essential for the maintenance of B cells through the regulation status of the JNK-mediated apoptosis signal (Xing et al., 2008). Significantly, our patient also presented the decreased CD4^+^ T lymphocytes and increased NK cells, and this condition suggested that PPP2R3C plays a role in the survival of multiple lymphocytes and identified *PPP2R3C* variants as a potential cause of the disorder of immune cells.

## Conclusion

In conclusion, novel compound heterozygous variants in *PPP2R3C* cause specific syndromic 46, XY gonadal dysgenesis, which broadened the pathogenic variant spectrum of *PPP2R3C*. The typical phenotype of *PPP2R3C* mutation is complete gonadal dysgenesis with multiple extragonadal anomalies, including facial deformities, skeletal system abnormalities, muscle abnormalities, impaired nervous system, impaired hearing and vision, heart and kidney anomalies, and gastrointestinal dysfunction. In addition, we found a decreased number of CD19^+^ B cells and CD4^+^ T cells in our patient, which is a new phenotype in syndromic 46, XY gonadal dysgenesis.

## Data Availability

The datasets for this article are not publicly available due to concerns regarding participant/patient anonymity. Requests to access the datasets should be directed to the corresponding authors.

## References

[B1] Altunoglu U. Börklü E. Shukla A. Escande‐Beillard N. Ledig S. Azaklı H. (2022). Expanding the Spectrum of Syndromic PPP2R3C ‐Related XY Gonadal Dysgenesis to XX Gonadal Dysgenesis. Clin. Genet. 101 (2), 221–232. 10.1111/cge.14086 34750818

[B2] Bagheri-Fam S. Ono M. Li L. Zhao L. Ryan J. Lai R. (2015). FGFR2mutation in 46,XY Sex Reversal with Craniosynostosis. Hum. Mol. Genet. 24 (23), 6699–6710. 10.1093/hmg/ddv374 26362256 PMC4634374

[B3] Bashamboo A. McElreavey K. (2015). Human Sex-Determination and Disorders of Sex-Development (DSD). Seminars Cell Dev. Biol. 45, 77–83. 10.1016/j.semcdb.2015.10.030 26526145

[B4] Baxter R. M. Arboleda V. A. Lee H. Barseghyan H. Adam M. P. Fechner P. Y. (2015). Exome Sequencing for the Diagnosis of 46,XY Disorders of Sex Development. J. Clin. Endocrinol. Metab. 100 (2), E333–E344. 10.1210/jc.2014-2605 25383892 PMC4318895

[B5] Cicek D. Warr N. Yesil G. Kocak Eker H. Bas F. Poyrazoglu S. (2021). Broad-Spectrum XX and XY Gonadal Dysgenesis in Patients with a Homozygous L193S Variant in PPP2R3C. Eur. J. Endocrinol. 186 (1), 65–72. 10.1530/EJE-21-0910 34714774 PMC8679844

[B6] Guran T. Yesil G. Turan S. Atay Z. Bozkurtlar E. Aghayev A. (2019). PPP2R3C Gene Variants Cause Syndromic 46,XY Gonadal Dysgenesis and Impaired Spermatogenesis in Humans. Eur. J. Endocrinol. 180 (5), 291–309. 10.1530/eje-19-0067 30893644

[B7] Hiorta O. Gillessen-Kaesbachb G. (2009). Disorders of Sex Development in Developmental Syndromes. Endocr. Dev. 14, 174–180. 10.1159/000207486 19293584

[B8] Malki S. Nef S. Notarnicola C. Thevenet L. Gasca S. Méjean C. (2005). Prostaglandin D2 Induces Nuclear Import of the Sex-Determining Factor SOX9 via its cAMP-PKA Phosphorylation. EMBO J. 24 (10), 1798–1809. 10.1038/sj.emboj.7600660 15889150 PMC1142593

[B9] Tamura M. Isojima T. Kasama T. Mafune R. Shimoda K. Yasudo H. (2017). Novel DHCR7 Mutation in a Case of Smith-Lemli-Opitz Syndrome Showing 46,XY Disorder of Sex Development. Hum. Genome Var. 4, 17015. 10.1038/hgv.2017.15 28503313 PMC5425407

[B10] Xing Y. Igarashi H. Wang X. Sakaguchi N. (2005). Protein Phosphatase Subunit G5PR Is Needed for Inhibition of B Cell Receptor-Induced Apoptosis. J. Exp. Med. 202 (5), 707–719. 10.1084/jem.20050637 16129705 PMC2212881

[B11] Xing Y. Wang X. Igarashi H. Kawamoto H. Sakaguchi N. (2008). Protein Phosphatase Subunit G5PR that Regulates the JNK-Mediated Apoptosis Signal Is Essential for the Survival of CD4 and CD8 Double-Positive Thymocytes. Mol. Immunol. 45 (7), 2028–2037. 10.1016/j.molimm.2007.10.028 18022237

[B12] Yu B. Q. Liu Z. X. Gao Y. J. Wang X. Mao J. F. Nie M. (2021). Prevalence of Gene Mutations in a Chinese 46,XY Disorders of Sex Development Cohort Detected by Targeted Next-Generation Sequencing. Asian J. Androl. 23 (1), 69–73. 10.4103/aja.aja_36_20 32985417 PMC7831832

